# Competition between cyanobacteria and green algae at low *versus* elevated CO_2_: who will win, and why?

**DOI:** 10.1093/jxb/erx027

**Published:** 2017-02-16

**Authors:** Xing Ji, Jolanda M H Verspagen, Maayke Stomp, Jef Huisman

**Affiliations:** Department of Aquatic Microbiology, Institute for Biodiversity and Ecosystem Dynamics, University of Amsterdam, GE Amsterdam, The Netherlands

**Keywords:** Carbon dioxide, climate change, competition model, CO_2_-concentrating mechanism, cyanobacteria, green algae, harmful algal blooms, lakes, *Microcystis aeruginosa*

## Abstract

Traditionally, it has often been hypothesized that cyanobacteria are superior competitors at low CO_2_ and high pH in comparison with eukaryotic algae, owing to their effective CO_2_-concentrating mechanism (CCM). However, recent work indicates that green algae can also have a sophisticated CCM tuned to low CO_2_ levels. Conversely, cyanobacteria with the high-flux bicarbonate uptake system BicA appear well adapted to high inorganic carbon concentrations. To investigate these ideas we studied competition between three species of green algae and a *bicA* strain of the harmful cyanobacterium *Microcystis aeruginosa* at low (100 ppm) and high (2000 ppm) CO_2_. Two of the green algae were competitively superior to the cyanobacterium at low CO_2_, whereas the cyanobacterium increased its competitive ability with respect to the green algae at high CO_2_. The experiments were supported by a resource competition model linking the population dynamics of the phytoplankton species with dynamic changes in carbon speciation, pH and light. Our results show (i) that competition between phytoplankton species at different CO_2_ levels can be predicted from species traits in monoculture, (ii) that green algae can be strong competitors under CO_2_-depleted conditions, and (iii) that bloom-forming cyanobacteria with high-flux bicarbonate uptake systems will benefit from elevated CO_2_ concentrations.

## Teaser

Contrary to the current paradigm, competition experiments showed that green algae defeated cyanobacteria at low CO_2_ levels, whereas cyanobacteria with high-flux carbon uptake systems became stronger competitors at elevated CO_2_.

## Introduction

Cyanobacterial blooms have become a major water quality problem in many eutrophic lakes worldwide (Chorus and Bartram, 1999; Verspagen *et al.*, 2006; Guo, 2007; Michalak *et al.*, 2013). They produce taste and odor compounds that may interfere with the recreational function of lakes and the preparation of drinking water (Chorus and Bartram, 1999; Watson *et al.*, 2008). Moreover, cyanobacteria can produce a variety of toxins, causing liver, digestive and neurological diseases when ingested by waterfowl, pets, cattle, and humans (Carmichael, 2001; Codd *et al.*, 2005; Huisman *et al.*, 2005; Merel *et al.*, 2013). Hence, an improved understanding of the environmental conditions that favor the dominance of cyanobacteria over eukaryotic phytoplankton species is desirable.

Dense cyanobacterial blooms often deplete the dissolved CO_2_ concentration in surface waters, sometimes down to less than 0.1 μmol l^–1^ corresponding to pCO_2_ less than 3 parts per million (ppm) (Lazzarino *et al.*, 2009; Balmer and Downing, 2011). CO_2_ depletion by dense blooms induces high pH values, above 9 or even 10 (Talling, 1976; Ibelings and Maberly, 1998; Verspagen *et al.*, 2014*b*). At these pH values, most dissolved inorganic carbon (DIC) is in the form of bicarbonate, and with increasing pH an increasing fraction of DIC is converted to carbonate. Under these conditions, CO_2_ availability can become an important limiting factor for photosynthesis. Cyanobacteria have developed a highly efficient CO_2_-concentrating mechanism (CCM) to take up CO_2_ and bicarbonate as inorganic carbon (C_i_) source, and to augment the intracellular CO_2_ level around the ribulose-1,5-bisphosphate carboxylase/oxygenase (RuBisCO) enzyme responsible for carbon fixation (Price *et al.*, 2008; Raven *et al.*, 2012; Burnap *et al.*, 2015). It has therefore been hypothesized that cyanobacteria are superior competitors at low CO_2_ levels, and will dominate waters in which the dissolved CO_2_ concentration has been depleted (Shapiro, 1990, 1997). Conversely, eukaryotic phytoplankton might be better competitors at high CO_2_ levels. This classic paradigm has received support from several competition experiments between cyanobacteria and green algae (Caraco and Miller, 1998; Low-Décarie *et al.*, 2011, 2015; but see Verschoor *et al.*, 2013). If this paradigm is true, the logical corollary is that rising CO_2_ levels will particularly benefit eukaryotic phytoplankton species at the expense of cyanobacteria.

Yet, recent insights indicate that this classic paradigm might be too simple. Although the details of the eukaryotic CCM are not yet fully understood, green algae can also deploy a sophisticated CCM well adapted to low CO_2_ levels (Moroney and Ynalvez, 2007; Wang *et al.*, 2011; Meyer and Griffiths, 2013). Furthermore, recent studies have revealed a striking genetic and phenotypic diversity in the CCM of harmful cyanobacteria (Sandrini *et al.*, 2014, 2015*b*; Visser *et al.*, 2016). All harmful freshwater cyanobacteria investigated so far contain two CO_2_ uptake systems and the ATP-dependent bicarbonate uptake system BCT1. In addition, however, some cyanobacteria deploy the high-affinity but low-flux bicarbonate uptake system SbtA, whereas other cyanobacteria deploy the low-affinity but high-flux bicarbonate uptake system BicA (Sandrini *et al.*, 2014). Therefore, three C_i_ uptake genotypes can be distinguished: (i) *sbtA* strains (high-affinity specialists), (ii) *bicA* strains (high-flux specialists), and (iii) *bicA*+*sbtA* strains (CCM generalists). These three genotypes were first described for the genus *Microcystis* (Sandrini *et al.*, 2014, 2016), but similar genetic diversity also exists within other harmful cyanobacterial genera such as *Dolichospermum* (formerly known as *Anabaena*) and *Planktothrix* (Visser *et al.*, 2016).

Laboratory selection experiments with mixtures of several *Microcystis* strains found that the strain composition shifted from *bicA*+*sbtA* strains at low pCO_2_ to *bicA* strains at high pCO_2_ (Sandrini *et al.*, 2016). Similarly, in a eutrophic lake, *bicA*+*sbtA* strains were dominant when C_i_ concentrations in the lake were depleted during a dense cyanobacterial bloom, but were replaced by *bicA* strains when C_i_ concentrations increased later in the season (Sandrini *et al.*, 2016). These results show that the genetic composition of cyanobacterial communities adapts to changes in C_i_ availability by means of natural selection, favoring CCM generalists at low CO_2_ levels while favoring high-flux specialists at high CO_2_. In natural waters, however, cyanobacteria compete not only amongst each other, but also against eukaryotic phytoplankton. How the competitive abilities of different cyanobacterial C_i_ uptake genotypes perform against eukaryotic species such as green algae has not yet been investigated.

Resource competition theory provides a theoretical framework to understand and predict how changes in resource availability may affect the species composition (Tilman, 1982; Huisman and Weissing, 1994; Grover, 1997). This body of theory uses the kinetic traits of species measured in monoculture to predict the dynamics and outcome of competition for limiting resources in species mixtures. Resource competition models have been successfully applied to predict competition for nutrients and light, both in qualitative and quantitative terms (Tilman, 1977; Sommer, 1985; Huisman *et al.*, 1999; Litchman *et al.*, 2004; Stomp *et al.*, 2004; Passarge *et al.*, 2006). Competition for inorganic carbon is conceptually more complicated, however, because the species compete for two resources (CO_2_ and bicarbonate) whose concentrations depend not only on resource uptake but also on pH and alkalinity. Moreover, pH and alkalinity depend in turn on a variety of biogeochemical processes and also change in response to the carbon and nutrient uptake activity by the phytoplankton community (Talling, 1976; Wolf-Gladrow *et al.*, 2007; Verspagen *et al.*, 2014*b*).

This study investigated competition between a harmful cyanobacterium (*Microcystis* PCC 7806) and three species of green algae (*Monoraphidium griffithii*, *Scenedesmus obliquus*, and *Chlorella vulgaris*) at low and at high CO_2_ concentrations. *Microcystis* PCC 7806 is a *bicA* strain (Sandrini *et al.*, 2014), and is therefore expected to be a relatively weak competitor at low CO_2_ levels but a stronger competitor at high CO_2_. To investigate this hypothesis, we ran monoculture experiments of each species at both low and high CO_2_ levels (100 and 2000 ppm). The results of these monoculture experiments were used to parameterize a resource competition model, which predicted the competitive interactions between the species based on dynamic changes in inorganic carbon chemistry, alkalinity and pH induced by the growing phytoplankton populations. Next, competition experiments were carried out to test the model predictions. Together, the theory and experiments may help in understanding shifts in phytoplankton community composition in response to rising CO_2_ levels.

## Competition model: theory and development

We first developed a model to investigate competition for inorganic carbon and light among phytoplankton species. The model combined previous theoretical work on growth and competition under light-limited (Huisman and Weissing, 1994; Huisman *et al.*, 1999; Passarge *et al.*, 2006) and carbon-limited conditions (Van de Waal *et al.*, 2011; Verspagen *et al.*, 2014*a*, *b*).

The model considers a well-mixed vertical water column, where depth *z* runs from 0 at the water surface to *z*_max_ at the bottom of the water column. The population dynamics of the phytoplankton species are governed by their light-dependent assimilation of carbon dioxide and bicarbonate. The model assumes eutrophic conditions, in which all nutrients are in ample supply and hence do not limit phytoplankton growth. Uptake of inorganic carbon and nutrients induces dynamic changes in pH and alkalinity. These changes in pH and alkalinity affect the availability of the different inorganic carbon species, which feeds back on phytoplankton growth. The growing phytoplankton populations also increase the turbidity of the water column, thereby diminishing the light available for further photosynthesis and growth.

### Species dynamics

The model assumes that the specific growth rates of the species depend on their carbon assimilation. Let *X*_*i*_ denote the population density of phytoplankton species *i*, and let *Q*_*i*_ denote its cellular carbon content (also known as carbon quota; sensu Droop, 1973). The population dynamics of a number of *n* competing species can then be written as:

$$\frac{\text{d}X_{i}}{\text{d}t}={\text{μ}}_{i}\left(Q_{i}\right)X_{i}-m_{i}X_{i}\;\;\;\;\;i=\text{1},\;\ldots ,n$$ (1)

where µ_*i*_(*Q*_*i*_) is the specific growth rate of species *i* as an increasing function of its carbon content, and *m*_*i*_ is its loss rate. We assume that each species requires a minimum cellular carbon content in order to function, and reaches its maximum specific growth rate when cells are satiated with carbon (see Supplementary Model S1 at *JXB* online). In our application, the loss rates of the species will be governed by the dilution rate of the chemostat (i.e. *m*_*i*_*=D*).

The carbon contents of the species increase through uptake of carbon dioxide (*u*_CO2,*i*_) and bicarbonate (*u*_HCO3,*i*_), and decrease through respiration (*r*_*i*_) and dilution of the carbon content by growth:

$$\frac{\text{d}Q_{i}}{\text{d}t}=u_{\text{CO}2,i}+u_{\text{HCO}3,i}-r_{i}-{\text{μ}}_{i}(Q_{i})Q_{i}\;\;\;\;i=\text{1},\;\ldots ,n$$ (2)

We assume that uptake rates of CO_2_ (*u*_CO2,*i*_) and bicarbonate (*u*_HCO3,*i*_) are increasing functions of the ambient CO_2_ and bicarbonate concentration according to Michaelis–Menten kinetics. Carbon uptake and assimilation require energy from the light reactions of photosynthesis, and therefore the carbon uptake rates also depend on the photosynthetic activity of the cells and hence on light availability. Furthermore, we incorporated a simple negative feedback loop in which carbon uptake rates decrease with increasing cellular carbon content, such that carbon uptake systems are active under carbon-limiting conditions (Eisenhut *et al.*, 2007; Burnap *et al.*, 2015; Wang *et al.*, 2015) and down-regulated when cells become satiated with carbon (Beardall and Giordano, 2002; Sandrini *et al.*, 2015*a*). Respiration rates (*r*_*i*_) of the species increase with their cellular carbon content, approaching maximum values when cells become satiated with carbon. The mathematical equations describing these relationships are presented in Supplementary Model S1.

### Light conditions

The underwater light gradient is described by the Lambert–Beer law (Huisman *et al.*, 1999):

$$I\left(z\right)=I_{\text{in}}\text{exp}(-K_{\text{bg}}z-\sum_{i=1}^{n}k_{i}X_{i}z)$$ (3)

where *I*(*z*) is the light intensity at depth *z*, *I*_in_ is the incident light intensity, *K*_bg_ is the background turbidity of the water itself, and *k*_*i*_ is the specific light attenuation coefficient of phytoplankton species *i*. We note that the light gradient changes dynamically, because the light intensity at a given depth decreases with increasing phytoplankton densities. We define *I*_out_ as the light intensity reaching the bottom of the water column (i.e. *I*_out_=*I*(*z*_max_)).

### Dissolved inorganic carbon

Changes in the concentration of total dissolved inorganic carbon, [DIC], are described by (Verspagen *et al.*, 2014*b*):

$$\frac{\text{d}\left[DIC\right]}{\text{d}t}=D\left({\left[DIC\right]}_{\text{in}}-\left[DIC\right]\right)+\frac{g_{CO2}}{z_{\text{max}}}-\overset{n}{\underset{i=1}{\sum }}\left(u_{CO2,i}+u_{HCO3,i}\right)X_{i}+\sum_{i=1}^{n}r_{i}X_{i}$$ (4)

The first term on the right-hand side of this equation describes changes through the influx ([DIC]_in_) and efflux of water containing DIC. The second term describes CO_2_ exchange with the atmosphere, where *g*_CO2_ is the CO_2_ flux across the air–water interface and division by *z*_max_ converts the flux per unit surface area into the corresponding change in DIC concentration. The third term describes uptake of dissolved CO_2_ and bicarbonate by the photosynthetic activity of the phytoplankton community. Finally, the fourth term describes CO_2_ release by respiration of the phytoplankton species.

The CO_2_ flux across the air–water interface, *g*_CO2_, depends on the difference in partial pressure. More specifically, *g*_CO2_ depends on the difference between the expected concentration of dissolved CO_2_ in water if in equilibrium with the partial pressure in the atmosphere and the actual dissolved CO_2_ concentration (Siegenthaler and Sarmiento, 1993; Cole *et al.*, 2010):

$$g_{\text{CO}2}=v\left(K_{0}{\text{pCO}}_{2}-\left[{\text{CO}}_{2}\right]\right)$$ (5)

where *v* is the gas transfer velocity (also known as piston velocity), *K*_0_ is the solubility of CO_2_ gas in water (also known as Henry’s constant), pCO_2_ is the partial pressure of CO_2_ in the atmosphere, and [CO_2_] is the dissolved CO_2_ concentration. In chemostats, gas transfer will depend on the gas flow rate (*a*). We therefore assume that *v*=*ba*, where *b* is a constant of proportionality.

Dissolved CO_2_, bicarbonate and carbonate concentrations, and pH were calculated from [DIC] and alkalinity (Stumm and Morgan, 1996). Assimilation of nitrate, phosphate and sulfate by phytoplankton involves proton consumption, thus increasing alkalinity (Wolf-Gladrow *et al.*, 2007; Verspagen *et al.*, 2014*b*). Therefore, the model treats alkalinity as a dynamic variable (see Supplementary Model S1 for details).

## Materials and methods

### Species

We performed monoculture and competition experiments with four freshwater phytoplankton species: the green algae *Monoraphidium griffithii* (Berk.) Kom.-Legn. (strain CCAP 202/15A), *Scenedesmus obliquus* (strain CCAP 276/3A) and *Chlorella vulgaris* Beyerinck (strain UTEX 259), and the cyanobacterium *Microcystis aeruginosa* (strain PCC 7806). *Microcystis* PCC 7806 produces the hepatotoxins microcystin-LR and [Asp^3^]-microcystin-LR and the potential neurotoxins cyanopeptolin A, C and 970 (Tonk *et al.*, 2009; Faltermann *et al.*, 2014). All four species were unialgal but not axenic. Regular microscopic inspection confirmed that concentrations of heterotrophic bacteria remained low throughout the experiments (<1% of the total biovolume measured with a CASY TTC cell counter; OLS OMNI Life Science, Bremen, Germany).

### Chemostat experiments

All experiments were conducted in laboratory-built chemostats, specifically designed to study the population dynamics of phytoplankton species (Huisman *et al.*, 2002; Passarge *et al.*, 2006; Verspagen *et al.*, 2014*b*). The chemostats allowed full control of light conditions, temperature, pCO_2_ in the gas flow, and nutrient concentrations in the mineral medium. Each chemostat consisted of a flat culture vessel with an optimal path length (‘mixing depth’) of *z*_max_=5 cm and a working volume of ~1.7 l. The vessel was illuminated from one side to create a unidirectional light gradient, using a constant incident light intensity (*I*_in_) of 50 ± 1 μmol photons m^–2^ s^–1^ provided by white fluorescent tubes (Philips PL-L 24W/840/4P, Philips Lighting, Eindhoven, The Netherlands). The temperature was maintained at 20 ± 1 °C with a stainless steel cooling finger inside each chemostat and connected to a Colora thermocryostat. To avoid nutrient limitation, the chemostats were provided with a very nutrient-rich mineral medium (Van de Waal *et al.*, 2009; Verspagen *et al.*, 2014*a*). The dilution rate was maintained at *D*=0.125 d^–1^.

### CO_2_ supply

We applied two CO_2_ treatments. The chemostats were bubbled with gas containing a CO_2_ concentration of either 100 ppm (‘low pCO_2_’) or 2000 ppm (‘high pCO_2_’) (Table 1). The gas was prepared as a mixture of pressurized air from which the variable CO_2_ concentration was completely removed using a CO_2_ scrubber (Ecodry K-MT6; Parker Zander, Lancaster, NY, USA) and subsequently a defined amount of pure CO_2_ gas was added to obtain the desired concentration using mass flow controllers (GT 1355R-2-15-A316 SS and 5850S, Brooks Instrument, Hatfield, PA, USA). Before entering the chemostats, the mixed gas was filter sterilized (0.2 µm Midisart 2000 Filter, Sartorius Stedim Biotech GmbH, Göttingen, Germany) and moisturized with Milli-Q water to suppress evaporation from the chemostats. The gas was dispersed as fine bubbles supplied from the bottom of the chemostat vessels at a constant flow rate of *a*=25 l h^–1^, which also ensured homogeneous mixing of the phytoplankton populations. We checked the CO_2_ concentration in the gas flow regularly using an environmental gas monitor (EGM-4; PP Systems, Amesbury, MA, USA).

**Table 1.** System parameters applied in the experiments

Parameter	Description	Value	Units
*D*	Dilution rate	0.125	d^–1^
*z*_max_	Mixing depth	0.05	m
*T*	Temperature	20	°C
*I*_in_	Incident light intensity	50	µmol photons m^–2^ s^–1^
*K*_bg_	Background turbidity^*a*^	7−11	m^–1^
DIC_in_	DIC concentration at influx^*b*^	0.5 and 2.0	mmol l^–1^
ALK_in_	Alkalinity at influx^*b*^	0.79 and 2.29	mEq l^–1^
sal	Salinity^*b*^	1.23 and 1.36	g l^–1^
pCO_2_	CO_2_ concentration in gas flow^*b*^	100 and 2000	ppm
*a*	Gas flow rate	25	l h^–1^
*v*	Gas transfer velocity^*b*^	0.24 and 0.68	m h^–1^

a Background turbidity varied among the chemostat vessels.

b The first value refers to the low pCO_2_ experiments and the second value to the high pCO_2_ experiments.

### Experimental measurements

The experiments were sampled at least every other day. The incident light intensity (*I*_in_) was measured with a LI-COR LI-250 quantum photometer (LI-COR Biosciences, Lincoln, NE, USA) at ten randomly chosen positions at the front surface of the chemostat vessel. Likewise, the light intensity transmitted through the chemostat (*I*_out_) was measured at the back surface of the chemostat vessel (Huisman *et al.*, 2002).

Population densities and biovolumes in samples of the monoculture experiments were measured in triplicate with a CASY TTC automated cell counter (OLS OMNI Life Science, Bremen, Germany) using a 60 µm capillary. The cell counter could not distinguish between the different species. Therefore, population densities in the competition experiments were counted on an Accuri C6 flow cytometer (Accuri Cytometers Inc., Ann Arbor, MI, USA), which distinguished the different species in these experiments on the basis of differences in pigmentation and cell size (side scatter). We did not perform competition experiments between *Scenedesmus* and *Chlorella*, because the flow cytometer could not adequately distinguish between these two chlorophytes.

Temperature and pH were measured immediately after sampling, using a SCHOTT pH meter (SCHOTT AG, Mainz, Germany). For DIC analysis, 35 ml samples were transferred to 50 ml falcon tubes, centrifuged for 15 min at 600 *g*, immediately filtered over 0.45 µm polyethersulfone membrane filters (Sartorius Stedim Biotech GmbH, Göttingen, Germany), transferred to gas-tight urine tubes (Terumo Europe NV, Leuven, Belgium) and stored at 4 ºC until analysis. DIC was analysed by phosphoric acid addition using a Model 700 TOC Analyzer (OI Corp., College Station, TX, USA). Concentrations of CO_2_(aq), bicarbonate and carbonate were calculated from DIC and pH, based on the dissociation constants of inorganic carbon corrected for temperature and salinity (Stumm and Morgan, 1996; Verspagen *et al.*, 2014*b*).

To determine the cellular carbon, nitrogen and sulfur content, pellets from the 50 ml falcon tube were transferred into 2 ml Eppendorf tubes, washed three times with a nutrient-free NaCl solution with a salinity equal to our medium, and stored at –20 °C. Subsequently, the pellets were freeze-dried and weighted, and the carbon, nitrogen and sulfur content of homogenized freeze-dried cell powder were analysed with a Vario EL Elemental Analyzer (Elementar Analysensysteme GmbH, Hanau, Germany).

### Model parameterization

The model parameters consisted of system parameters and species parameters. System parameters are under experimental control and were regularly measured during the experiments. We had already specified several of the system parameters, such as the incident light intensity (*I*_in_), pCO_2_ level in the gas flow, and dilution rate (*D*) of the chemostats. A complete list of all system parameters is provided in Table 1.

Species parameters describe the traits of the species. Some species parameters were measured experimentally. The maximum specific growth rate (µ_max,*i*_) and minimum cellular carbon content (*Q*_min,*i*_) were determined in batch cultures at an *I*_in_ of 50 ± 1 μmol photons m^–2^ s^–1^ and a temperature of 20 ± 1 ° C. Maximum specific growth rate was measured in batch cultures aerated with gas containing a saturating CO_2_ concentration of 10 000 ppm. The minimum cellular carbon content was measured as the cellular carbon content in unaerated dense batch cultures that were first grown for a day in medium to which no DIC was added, and were subsequently incubated overnight in the dark. The cellular N:C and S:C ratios (*c*_N,*i*_ and *c*_S,*i*_) were calculated from the cellular carbon, nitrogen and sulfur contents measured in the steady-state monocultures of the species. The specific light attenuation coefficients (*k*_*i*_) of the species were estimated from the monoculture experiments using the Lambert–Beer law. For monocultures, Eqn (3) can be written as ln(*I*_in_*/I*_out_)/*z*_max_=*K*_bg_+*k*_*i*_*X*_*i*_. Hence, the specific light attenuation coefficient (*k*_*i*_) was estimated as the slope of a linear regression of ln(*I*_in_*/I*_out_)/*z*_max_*versus* the population density *X*_*i*_, and the background turbidity (*K*_bg_) was estimated as the intercept.

All other species parameters were estimated by fitting the model predictions to the observed dynamics in the monoculture experiments. More specifically, we fitted the time courses of population density, light transmission (*I*_out_), pH and inorganic carbon concentrations predicted by the model to the time courses measured in the monoculture experiments, following the same methodology as in earlier studies (Huisman *et al.*, 1999; Passarge *et al.*, 2006; Verspagen *et al.*, 2014*b*). To avoid overfitting, the model was fitted simultaneously to both the low pCO_2_ and high pCO_2_ monocultures, resulting in eight parameter estimates per species.

The species parameters obtained from the monoculture experiments were combined with the system parameters to predict the population dynamics and inorganic carbon chemistry in the competition experiments.

In the competition experiments, we calculated the rate of competitive displacement (RCD) from the slope of the linear regression of ln(*X*_1_/*X*_2_) *versus* time, where *X*_1_ and *X*_2_ are the population densities of the two competing species (Grover, 1991; Passarge *et al.*, 2006).

## Results

### Monoculture experiments at low pCO_2_

**Fig. 1.**
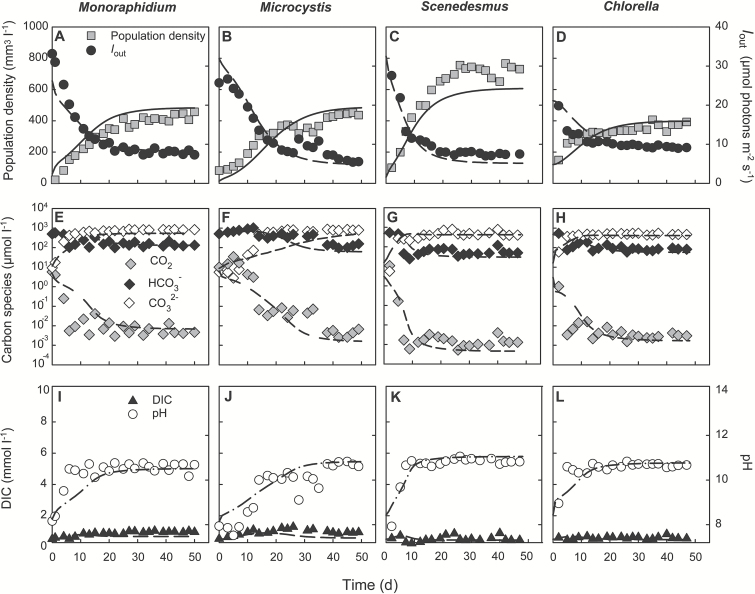
Monoculture experiments at low pCO_2_ (100 ppm). (A–D) Population density (expressed as biovolume) and light intensity *I*_out_ penetrating through the chemostat. (E–H) CO_2_(aq), bicarbonate and carbonate concentrations. (I–L) Dissolved inorganic carbon (DIC) and pH. Different panels represent different species: (A, E, I) *Monoraphidium*; (B, F, J) *Microcystis*; (C, G, K) *Scenedesmus*; (D, H, L) *Chlorella.* Symbols indicate experimental data, and lines indicate model fits. Parameter values of the model are provided in Table 1 and Table 2.

At low pCO_2_, the phytoplankton species increased until a steady state was reached with population densities (expressed as biovolumes) ranging from 370 mm^3^ l^–1^ for *Chlorella* to 720 mm^3^ l^–1^ for *Scenedesmus* (Fig. 1A–D; Supplementary Table S1). The growing phytoplankton populations reduced the light intensity penetrating through the chemostats (*I*_out_) to 6.2–9.3 µmol photons m^–2^ s^–1^ and depleted the dissolved CO_2_ concentration by several orders of magnitude to <0.01 μmol l^–1^ (Fig. 1E–H). Bicarbonate concentrations decreased about one order of magnitude, and were offset by a similar increase of the carbonate concentrations, such that the total DIC concentration in the chemostats remained more or less constant. Alkalinity increased to 1.3–2.0 mEq l^–1^ depending on the species (see Supplementary Table S1 at *JXB* online). The pH increased by more than two units, from initial values of ~8 to steady state values of ~10.7 (Fig. 1I–L). The relatively high values of *I*_out_ in combination with severe CO_2_ depletion and a high pH indicate that the phytoplankton growth rates in these experiments were carbon limited.

### Monoculture experiments at high pCO_2_

**Fig. 2.**
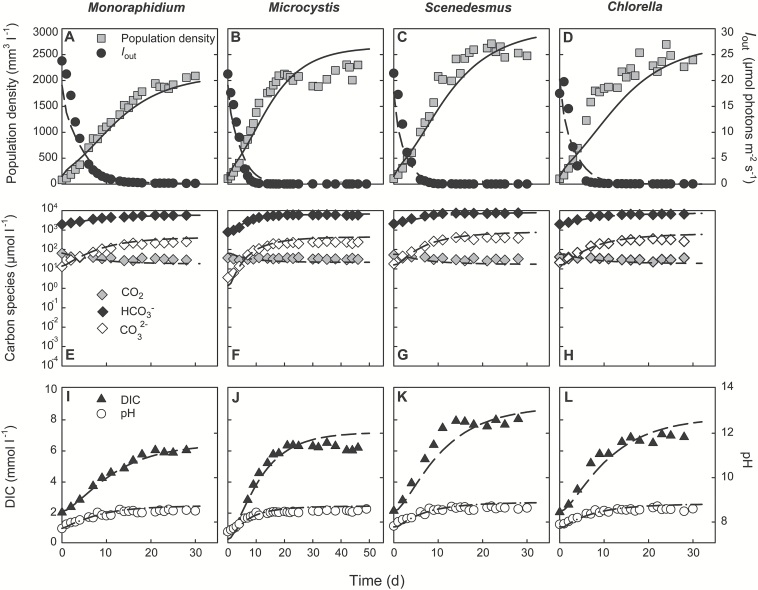
Monoculture experiments at high pCO_2_ (2000 ppm). (A–D) Population density (expressed as biovolume) and light intensity *I*_out_ penetrating through the chemostat. (E–H) CO_2_(aq), bicarbonate and carbonate concentrations. (I–L) Dissolved inorganic carbon (DIC) and pH. Different panels represent different species: (A, E, I) *Monoraphidium*; (B, F, J) *Microcystis*; (C, G, K) *Scenedesmus*; (D, H, L) *Chlorella.* Symbols indicate experimental data, and lines indicate model fits. Parameter values of the model are provided in Table 1 and Table 2.

At high pCO_2_, the phytoplankton species reached population densities ranging from ~2000 mm^3^ l^–1^ for *Monoraphidium* to ~2500 mm^3^ l^–1^ for *Scenedesmus* (Fig. 2A–D; Supplementary Table S1). These values are 3.5–6 times higher than in the low pCO_2_ experiments (Fig. 1A–D), thus confirming that phytoplankton growth in the low pCO_2_ experiments was indeed carbon limited. The dense phytoplankton populations absorbed almost all incident light, reducing the light intensity penetrating through the chemostats (*I*_out_) to <0.2 µmol photons m^–2^ s^–1^ (Fig. 2A–D). The dissolved CO_2_ concentration was only slightly reduced to ~30 μmol l^–1^ (Fig. 2E–H). By contrast, the bicarbonate and carbonate concentration and hence also the total DIC concentration increased during the experiments (Fig. 2I–L). The DIC increase was enabled by a rise in alkalinity, from 2.3 mEq l^–1^ at the start of the experiments to 6.2–8.4 mEq l^–1^ at steady state (see Supplementary Table S1). Alkalinity increased during the experiments, because the high uptake rates of nitrate, phosphate, and sulfate by the growing phytoplankton populations are accompanied by proton consumption to maintain charge balance (Goldman and Brewer, 1980; Wolf-Gladrow *et al.*, 2007). A similar increase in DIC and alkalinity induced by dense phytoplankton populations was also observed in earlier chemostat studies (Verspagen *et al.*, 2014*b*). The pH increased only slightly from ~8 to ~8.6 (Fig. 2I–L). The very low *I*_out_ values in combination with high dissolved CO_2_ and bicarbonate concentrations indicate that the phytoplankton growth rates in these experiments were light limited.

**Table 2.** Species parameters estimated from the monoculture experiments

Parameter	Description	*Monoraphidium*	*Microcystis*	*Scenedesmus*	*Chlorella*	Units
µ_max_	Maximum specific growth rate^*a*^	1.27 ± 0.05	1.04 ± 0.02	1.39 ± 0.05	1.28 ± 0.03	d^–1^
*k*	Specific light attenuation coefficient^*a*^	6 × 10^–5^±0.7 × 10^–6^	8 × 10^–5^±1.0 × 10^–6^	6 × 10^–5^±1.0 × 10^–6^	6 × 10^–5^±1.0 × 10^–6^	m^2^ mm^–3^
*H*	Half-saturation constant for light^*b*^	50	11	30	48	µmol photons m^–2^ s^–1^
*H*_CO2_	Half-saturation constant for CO_2_^*b*^	12.5	1.0	2.0	2.5	µmol l^–1^
*H*_HCO3_	Half-saturation constant for HCO_3_^–*b*^	80	20	30	40	µmol l^–1^
*r*_max_	Maximum respiration rate^*b*^	2.2	0.9	2.2	2.2	µmol mm^–3^ d^–1^
*u*_max,CO2_	Maximum uptake rate of CO_2_^*b*^	17.1	20.1	14.7	22.5	µmol mm^–3^ d^–1^
*u*_max,HCO3_	Maximum uptake rate of HCO_3_^–*b*^	14.0	10.8	14.7	15.7	µmol mm^–3^ d^–1^
*Q*_min_	Minimum cellular carbon content^*a*^	14.0 ± 1.1	9.0 ± 2.7	12.5 ± 1.0	11.5 ± 0.8	µmol mm ^–3^
*Q*_max_	Maximum cellular carbon content^*b*^	24.5	29.9	21.1	29.9	µmol mm ^–3^
*c*_N_	Cellular N:C ratio^*a*^	0.114 ± 0.002	0.130 ± 0.003	0.125 ± 0.003	0.100 ± 0.002	Molar ratio
*c*_P_	Cellular P:C ratio^*b*^	7.1 × 10^–3^	8.1 × 10^–3^	7.8 × 10^–3^	6.3 × 10^–3^	Molar ratio
*c*_S_	Cellular S:C ratio^*a*^	3.8 × 10^–3^±0.2 × 10^–3^	7.5 × 10^–3^±0.3 × 10^–3^	6.0 × 10^–3^±0.3 × 10^–3^	8.2 × 10^–3^±0.3 × 10^–3^	Molar ratio

a Parameter values measured experimentally, given as mean±standard error.

b Parameter values estimated by fitting the model predictions to time courses of the experiments.

### Predictions derived from the monoculture experiments

The monoculture experiments showed dynamic changes in population abundances, light conditions, carbon speciation, alkalinity, and pH, which caused concomitant changes in the growth rates of the species. We tried to capture these dynamics by the development of a mathematical model. The results show that the model generally fitted well to the monoculture data, in the experiments at both low pCO_2_ (Fig. 1) and high pCO_2_ (Fig. 2). The species parameters estimated from the monoculture experiments are summarized in Table 2, and will be used to predict the dynamics of the competition experiments.

Resource competition theory can provide some further insights. Consider several species competing for a single limiting resource. Each species has its own critical R*, defined as the resource availability at which the specific growth rate of a species equals its loss rate. During competition, resource availability diminishes as the resource is consumed by the species. One by one, species start to decline when resource availability is depleted below their R* values. This process continues, until eventually the species with lowest R* has competitively displaced all other species. Hence, resource competition theory predicts that the species with lowest R* will be the superior competitor (Tilman, 1982; Grover, 1997).

**Fig. 3.**
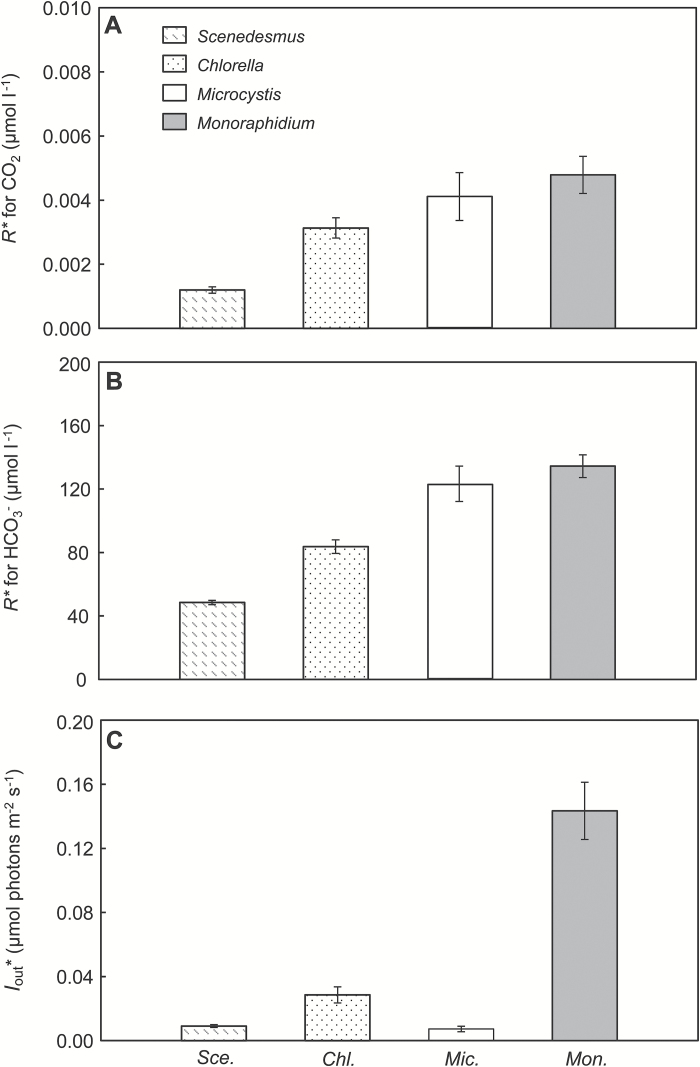
R* values of the species. (A, B) R* values for CO_2_(aq) (A) and bicarbonate (B) of the species, indicative of their competitive abilities for inorganic carbon. R* values were estimated from the CO_2_ and bicarbonate concentrations measured in the steady-state monocultures at low pCO_2_. (C) Critical light intensities (I*_out_) of the species, indicative of their competitive abilities for light. Critical light intensities were estimated from the light intensities penetrating through the steady-state monocultures at high pCO_2_. All estimates are based on the mean±SD of the last five data points of each monoculture experiment. Sce, *Scenedesmus*; Chl, *Chlorella*; Mic, *Microcystis*; Mon, *Monoraphidium*.

In many applications, the R* value of each species is measured as the steady-state concentration of the limiting resource in monoculture (e.g. Tilman, 1981; Passarge *et al.*, 2006; Wilson *et al.*, 2007). In our application, however, CO_2_ and bicarbonate provide two alternative inorganic carbon sources that are rapidly interconverted by the chemical reaction of CO_2_ with water, which makes it difficult to measure the R* for CO_2_ and R* for bicarbonate independently. Nevertheless, the steady-state concentrations of CO_2_ and bicarbonate in carbon-limited monoculture may provide useful information on the competitive abilities for inorganic carbon of the species. That is, if we assume that a low steady-state CO_2_ concentration in monoculture implies a high competitive ability for CO_2_, then the species can be ranked according to their competitive ability for CO_2_ as (Fig. 3A): *Scenedesmus* > *Chlorella* > *Microcystis* > *Monoraphidium*. Similarly, based on the steady-state bicarbonate concentrations, the species can be ranked according to their competitive ability for bicarbonate as (Fig. 3B): *Scenedesmus* > *Chlorella* > *Microcystis* > *Monoraphidium*. Accordingly, the ranking of the species is the same for both CO_2_ and bicarbonate, indicating that *Scenedesmus* will be the best competitor for inorganic carbon, followed by *Chlorella* and then *Microcystis*, while *Monoraphidium* is the worst competitor for inorganic carbon.

At high CO_2_ levels, CO_2_ and bicarbonate were in ample supply, but light becomes a limiting resource (Fig. 2A–D). Analogous to R*, competition theory predicts that the species with lowest critical light intensity (I*_out_) is the superior competitor for light (Huisman and Weissing, 1994; Huisman *et al.*, 1999). The critical light intensities of the species were measured as the steady-state values of *I*_out_ in the monoculture experiments at high pCO_2_. Based on their critical light intensities, the species can be ranked according to their competitive ability for light as (Fig. 3C): *Microcystis* ≈ *Scenedesmus* > *Chlorella* > *Monoraphidium*. Hence, from the monoculture data, *Microcystis* and *Scenedesmus* are predicted to be the best competitors for light with an approximately similar competitive ability, followed by *Chlorella*, while *Monoraphidium* is the worst competitor for light.

Comparison of the above species rankings indicates that *Microcystis* will become a stronger competitor when the species interactions shift from competition for inorganic carbon at low pCO_2_ to competition for light at high pCO_2_. The relative ranking among the three green algae remains unaltered with rising pCO_2_.

### Competition experiments

**Fig. 4.**
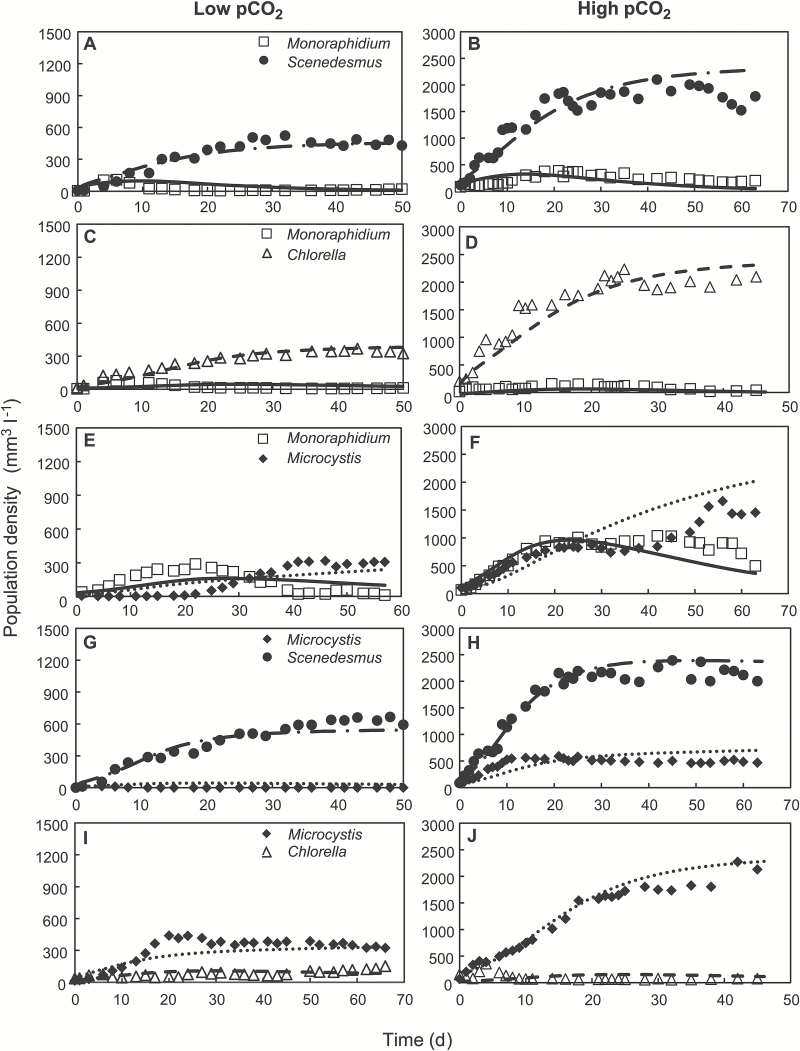
Competition experiments. The competition experiments were performed at low pCO_2_ (100 ppm; left panels) and high pCO_2_ (2000 ppm; right panels). (A, B) Competition between *Monoraphidium* and *Scenedesmus*. (C, D) Competition between *Monoraphidium* and *Chlorella*. (E, F) Competition between *Monoraphidium* and *Microcystis*. (G, H) Competition between *Microcystis* and *Scenedesmus*. (I, J) Competition between *Microcystis* and *Chlorella*. Dynamic changes in light conditions, carbon speciation and pH during the competition experiments are presented in Supplementary Figs S1 and S2. Symbols indicate experimental data, and lines indicate model predictions. Parameter values of the model are provided in Tables 1 and 2.

The competition experiments largely confirmed the predictions derived from the monoculture experiments. For instance, in the competition experiment at low pCO_2_ between *Monoraphidium* and *Scenedesmus*, both species increased during the first 6 d (Fig. 4A). Meanwhile, pH increased to 10.8 (see Supplementary Fig. S1A), the dissolved CO_2_ concentration was depleted to <0.004 μmol l^–1^ and the bicarbonate concentration decreased to ~100 μmol l^–1^ (Supplementary Fig. S2A). These concentrations are below the R* values for CO_2_ and bicarbonate of *Monoraphidium* (Fig. 3A, B). Hence, as predicted, *Monoraphidium* started to decline after the first week, and was competitively displaced by *Scenedesmus* (Fig. 4A).

At low pCO_2_, *Monoraphidium* was also outcompeted by *Chlorella* (Fig. 4C) and *Microcystis* (Fig. 4E). Hence, *Monoraphidium* was the weakest competitor. Furthermore, in line with expectation, *Scenedesmus* competitively displaced *Microcystis* (Fig. 4G). Contrary to expectation, *Chlorella* and *Microcystis* appeared to coexist at low pCO_2_ (Fig. 4I). During the last 20 d of this experiment, however, *Chlorella* tended to increase slowly at the cost of *Microcystis*, although the experiment didn’t last long enough to witness the final outcome. The latter result is in agreement with the rate of competitive displacement (Table 3), which indicated that *Chlorella* was indeed slowly displacing *Microcystis*. According to the competition experiments, the competitive ranking of the species at low pCO_2_ can thus be summarized as follows: *Scenedesmus* > *Chlorella* ≥ *Microcystis* > *Monoraphidium*. This is in good agreement with the rankings based on the R* values for CO_2_ and bicarbonate estimated in the monoculture experiments.

**Table 3.** Rates of competitive displacement (RCD±standard error) in the competition experiments RCD was calculated as the slope of a linear regression of ln(*X*_1_/*X*_2_) *versus* time, where *X*_1_ and *X*_2_ are the population densities of species 1 and species 2. The coefficient of determination (*R*^2^), number of data points (*n*) and significance (*P*) of the linear regression are indicated.

Competition experiment	RCD (d^–1^)	*R* ^2^	*n*	*P*
*Monoraphidium vs Microcystis*				
Low pCO_2_	–0.250 ± 0.008	0.99	14	<0.001
High pCO_2_	–0.043 ± 0.005	0.88	11	<0.001
*Monoraphidium vs Scenedesmus*				
Low pCO_2_	–0.292 ± 0.021	0.97	8	<0.001
High pCO_2_	–0.021 ± 0.002	0.84	20	<0.001
*Monoraphidium vs Chlorella*				
Low pCO_2_	–0.100 ± 0.008	0.93	16	<0.001
High pCO_2_	–0.062 ± 0.008	0.82	15	<0.001
*Scenedesmus vs Microcystis*				
Low pCO_2_	+0.654 ± 0.071	0.95	6	<0.001
High pCO_2_	0.000 ± 0.003	0.00	11	n.s.
*Microcystis vs Chlorella*				
Low pCO_2_	–0.039 ± 0.071	0.96	11	<0.001
High pCO_2_	+0.197 ± 0.025	0.92	11	<0.001

In their competition experiment at high pCO_2_, both *Monoraphidium* and *Scenedesmus* increased during the first 20 d (Fig. 4B). Meanwhile, pH stabilized at ~8.5 (see Supplementary Fig. S1B at *JXB* online), the dissolved CO_2_ concentration slightly decreased to ~20 μmol l^–1^ and the bicarbonate concentration increased to >3,000 μmol l^–1^ (Supplementary Fig. S2B). However, the incident light was almost completely absorbed by the dense species mixture, with <0.1 µmol photons m^–2^ s^–1^ penetrating through the chemostat (Supplementary Fig. S1B). This value is below the critical light intensity of *Monoraphidium* (Fig. 3C). Hence, after the first 20 d, *Monoraphidium* declined, and was gradually displaced by *Scenedesmus* (Fig. 4B).

At high pCO_2_, *Monoraphidium* was also outcompeted by *Chlorella* (Fig. 4D) and *Microcystis* (Fig. 4F), and hence it was again the weakest competitor. Interestingly, *Scenedesmus* and *Microcystis* appeared to coexist (Fig. 4H), in agreement with the similar critical light intensities of these two species (Fig. 3C). Their coexistence at high pCO_2_ was confirmed by the rate of competitive displacement, which did not differ significantly from zero for this species pair (Table 3). In line with expectation, *Microcystis* competitively displaced *Chlorella* (Fig. 4J). Hence, according to the competition experiments, the competitive abilities of the species at high pCO_2_ can be ranked as follows: *Microcystis* ≈ *Scenedesmus* > *Chlorella* > *Monoraphidium*.

This matches the species ranking based on their critical light intensities in monoculture.

Also quantitatively, the population dynamics predicted by the competition model agreed well with the results of the competition experiments (compare lines *versus* symbols in Fig. 4, and Supplementary Figs S1 and S2), both at low pCO_2_ and at high pCO_2_.

## Discussion

### Cyanobacteria *versus* green algae

Both our model predictions and our experimental results contradict the classic view (Shapiro, 1990; Caraco and Miller, 1998; Low-Décarie *et al.*, 2011, 2015) that cyanobacteria are strong competitors at low CO_2_ levels, whereas eukaryotic phytoplankton such as green algae are better competitors at elevated CO_2_. We found the opposite. At low CO_2_ levels, the cyanobacterium *Microcystis* was a relatively poor competitor. It lost in competition with *Scenedesmus*, was slowly replaced by *Chlorella*, and won only against *Monoraphidium*. At high CO_2_ levels, *Microcystis* was a stronger competitor. It won in competition with both *Monoraphidium* and *Chlorella*, and coexisted with *Scenedesmus*. *Microcystis* was the only species that increased its competitive ranking at elevated CO_2_; the relative ranking among the three species of green algae did not change.

### Competition at low CO_2_

In the competition experiments at low pCO_2_, the growing phytoplankton populations depleted the dissolved CO_2_ concentration within the first 1–3 weeks of the experiments and also the bicarbonate concentration declined. As a consequence, the growth rates of the phytoplankton populations slowed down, and one of the species in the experiments started to displace the other. Interestingly, both the ranking of the R* values estimated from the monocultures and the competitive replacements observed in the competition experiments show that the green algae *Scenedesmus* and *Chlorella* were stronger competitors for inorganic carbon than the cyanobacterium *Microcystis*.

These results can to a large extent be explained by the CCMs of the species. In particular, the cyanobacterium *Microcystis* PCC 7806 used in this study is a *bicA* strain (*sensu*Sandrini *et al.*, 2014). It contains the bicarbonate uptake systems BicA and BCT1, but lacks the high-affinity bicarbonate uptake system SbtA. Batch experiments have shown that *Microcystis* PCC 7806 has a lower growth rate at low C_i_ levels than strains that do have SbtA (Sandrini *et al.*, 2014). BCT1 is induced when *Microcystis* PCC 7806 grows at low CO_2_ levels (Sandrini *et al.*, 2015*a*, *b*), but this uptake system appears to have a slightly lower affinity for bicarbonate than SbtA, at least in the cyanobacteria *Synechocystis* PCC 6803 and *Synechococcus* PCC 7002 (Price *et al.*, 2004, 2008). Moreover, bicarbonate uptake by BCT1 is ATP dependent and therefore energetically quite expensive. The Na^+^-dependent bicarbonate uptake system BicA has a high flux rate but low affinity for bicarbonate (Price *et al.*, 2004). Hence, the lack of SbtA offers a plausible explanation of why *Microcystis* PCC 7806 had a selective disadvantage under C_i_-limited conditions. Previous experiments have indeed shown that such *bicA* strains were selectively displaced by *bicA*+*sbtA* strains under C_i_-limited conditions (Sandrini *et al.*, 2016). Our results show that cyanobacteria that lack the high-affinity bicarbonate uptake system SbtA are relatively poor competitors under C_i_-limited conditions, not only in comparison with other cyanobacteria but also in comparison with green algae such as *Scenedesmus* and *Chlorella*.

Our results do not of course imply that cyanobacteria are generally poor competitors under C_i_-limited conditions. *Microcystis* strains containing the high-affinity bicarbonate uptake system SbtA sustain higher growth rates at low C_i_ concentrations than *bicA* strains such as *Microcystis* PCC 7806 (Sandrini *et al.*, 2014). Moreover, recent selection experiments and lake data show that *bicA*+*sbtA* strains have a competitive advantage over *bicA* strains at low CO_2_ levels (Sandrini *et al.*, 2016). Whether SbtA-containing cyanobacteria or green algae such as *Scenedesmus* and *Chlorella* are better competitors at low CO_2_ levels thus remains an interesting open question.

Compared with cyanobacteria, less is known about the functioning of the CCMs in green algae. Our current understanding of eukaryotic CCMs comes largely from studies with the model organism *Chlamydomonas reinhardtii* (Moroney and Ynalvez 2007; Spalding, 2008; Wang *et al.*, 2011; Meyer and Griffiths, 2013). Briefly, the key components of the eukaryotic CCM are quite similar to the cyanobacterial CCM and involve active CO_2_ and bicarbonate uptake, interconversion between these two C_i_ species by carbonic anhydrases (CAs), and a microcompartment that contains RuBisCO (Wang *et al.*, 2015). In *Chlamydomonas*, five C_i_ transporters have been localized, including two confirmed bicarbonate transporters (HLA3 on the plasma membrane and LCIA on the chloroplast envelope) and three undefined C_i_ transporters (LCIl on the plasma membrane and CCAP1/2 on the chloroplast envelope). Inside the chloroplast, RuBisCO is densely packed in a microcompartment named the pyrenoid (Kuchitsu *et al.*, 1988; Engel *et al.*, 2015). Within the pyrenoid, bicarbonate is dehydrated to CO_2_ by a CA and released to RuBisCO (Moroney and Ynalvez, 2007).

So far, there are no studies on the CCM genes of the green algal strains we used in this study, but there is some recent work on the CCM genes of a different species of *Chlorella*, *C. pyrenoidosa*, indicating that this species has a CCM similar to that of *Chlamydomonas* (Fan *et al.*, 2016). For instance, when shifting *C. pyrenoidosa* from high CO_2_ to low CO_2_ conditions, CCM-related genes such as *LCIA*, *LCIB*, and *HLA3* showed increased expression, similar to the response of *C. reinhardtii* (Fan *et al.*, 2015). Interestingly, earlier work indicates that there is substantial variation in CCM activity within the *Chlorella* genus and even between different strains of the same species of *Chlorella*. For example, *C. vulgaris* strain 11 h and strain UTEX 263 seem to utilize only CO_2_ as a carbon source (Miyachi *et al.*, 1983, Tu *et al.*, 1986), whereas *C. vulgaris* strain C-3, strain UTEX 259 and *C. pyrenoidosa* can use both CO_2_ and bicarbonate (Miyachi *et al.*, 1983). Our results indicate that the *C. vulgaris* strain we used can utilize bicarbonate: if this green alga could only use CO_2_, it would not be able to grow in monoculture in our chemostats at a dilution rate of 0.125 d^–1^ and dissolved CO_2_ concentrations lower than 0.01 µmol l^–1^ (Fig. 1).

Similarly, the fact that *S. obliquus* and *M. griffithii* were also able to grow at CO_2_ concentrations below 0.01 µmol l^–1^ in our chemostat experiments indicates that these green algae can also utilize bicarbonate as carbon source. Earlier studies confirm the presence of a functional CCM in a different strain of *S. obliquus*. WT strain D3 has increased intracellular and extracellular CA activity, as well as a higher affinity for the uptake of CO_2_ and bicarbonate at low compared with high CO_2_ concentrations (Palmqvist *et al.*, 1994). Furthermore, in our experiments *S. obliquus* could deplete dissolved CO_2_ and bicarbonate concentrations to even lower levels than *Microcystis* PCC 7806 (Fig. 3), and displace *Microcystis* in the competition experiments at low CO_2_ (Fig. 4). Similar results were obtained in CO_2_-limited mesocosm experiments by Verschoor *et al.* (2013), where the same strain of *S. obliquus* displaced *Synechocystis* PCC 6803, a cyanobacterium that contains all five C_i_ uptake systems (Badger *et al.*, 2006). So far, very little is known about the CCM of *Monoraphidium griffithii*, but a related species, *M. braunii*, can photoactivate a blue-light-dependent bicarbonate transport system under CO_2_-limiting conditions (Mora *et al* 2002).

In total, these physiological studies indicate that many green algae are well adapted to cope with C_i_ limitation. Similar to cyanobacteria, many species of green algae are able to induce a CCM under CO_2_-limiting conditions (Meyer and Griffiths, 2013). Furthermore, in eutrophic lakes, not only cyanobacteria but also green algae have been observed to develop dense blooms at low CO_2_ concentrations and high pH (Jeppesen *et al.*, 1990; Jensen *et al.*, 1994; Beklioglu and Moss, 1995). The results of our competition experiments are in agreement with these physiological studies and field observations, and demonstrate that green algae can indeed be very effective competitors at low CO_2_ levels.

### Competition at elevated CO_2_

The notion that *Microcystis* PCC 7806 lacks the high-affinity uptake system SbtA explains not only why it was a poor competitor at low CO_2_ levels, but may also help to understand why it was more successful at high CO_2_ levels. In *bicA*+*sbtA* strains of *Microcystis*, the bicarbonate uptake genes *bicA* and *sbtA* are located on the same operon and are co-transcribed (Sandrini *et al.*, 2014). Transcription of *sbtA* will be inefficient and costly, however, when inorganic concentrations are high and hence the high-affinity but low-flux system SbtA is no longer needed. This reasoning is supported by recent selection experiments, which have shown that *bicA* strains have a competitive advantage over *bicA*+*sbtA* strains at elevated CO_2_ levels (Sandrini *et al.*, 2016).

Yet, the presence of the high-flux bicarbonate uptake system BicA is probably not sufficient to explain its competitive success at elevated CO_2_ levels. Elevated CO_2_ increased the dissolved C_i_ concentrations, but also yielded 3- to 8-fold higher population densities, which in turn generated very low light availabilities (Figs 1 and 2, and Supplementary Table S1). At these high CO_2_ but low light levels, carbon availability is no longer a major limiting factor and bicarbonate uptake systems tend to be down-regulated (Beardall, 1991; Sandrini *et al.*, 2015*a*). Instead, the intense shading induced by the dense phytoplankton populations will favor species adapted to low light environments (Huisman *et al.*, 1999). A range of studies have suggested that cyanobacteria are superior competitors for light (Mur *et al.*, 1977; Reynolds *et al.*, 1987; Huisman *et al.*, 2004; Yang and Jin, 2008; Schwaderer *et al.*, 2011). Comparison of the traits of the species investigated in this study shows that *Microcystis* PCC 7806 had the lowest half-saturation constant for light-limited growth (Table 2), indicating that it can sustain a relatively high growth rate at low light levels in comparison with the other species. *Scenedesmus* also had a relatively low half-saturation constant for light-limited growth, albeit higher than *Microcystis*, and had the highest maximum growth rate of all four species (Table 2). Indeed, the competitive ability of the cyanobacterium *Microcystis* increased at elevated CO_2_, and together with *Scenedesmus* it became the best competitor for light (Fig. 3).

### Model predictions

Comparison of the model predictions and experimental results shows that the impact of elevated CO_2_ on phytoplankton competition can be quite accurately predicted under controlled laboratory conditions. First, the system parameters and several species parameters were measured in monoculture experiments, while the remaining species parameters were estimated from least-squares fits of the model predictions to the monoculture dynamics (Figs 1 and 2). Hence, the model is calibrated with the monoculture data. Subsequently, the parameter estimates from the monocultures were used to predict the time course and outcome of competition in the species mixtures. Accordingly, the model predictions are validated with the competition experiments (Fig. 4, and Supplementary Figs S1 and S2).

Our model and experiments are of course still a major simplification in comparison with the complexities of the real CCMs of cyanobacteria and green algae competing in natural waters. For instance, the model brushes over many of the physiological details involved in the regulation of CCMs in response to changes in carbon and light availability (e.g. Beardall and Giordano, 2002; Burnap *et al.*, 2015). Furthermore, the experiments were limited to only a small number of phytoplankton species under controlled laboratory conditions, in isolation from a multitude of other hydrological, biogeochemical and ecological processes that are important in lakes. Species might be superior competitors for inorganic carbon or light, but if they are also preferentially grazed by zooplankton they are still unlikely to gain dominance in natural waters. Hence, further improvement of the model predictions might be obtained by further refinement of the underlying physiological and ecological processes.

Nevertheless, the results are promising. Our model and experiments seemed to capture the basic ingredients required to predict phytoplankton competition at different CO_2_ levels. Similar to several previous studies (e.g. Caraco and Miller, 1998; Low-Décarie *et al.*, 2011; Trimborn *et al.*, 2013), we have shown that species traits measured in monoculture can be used to predict changes in phytoplankton species composition at elevated CO_2_. To our knowledge, our study is the first experimental demonstration that a mathematical model can quantitatively predict dynamic changes in phytoplankton species composition, carbon speciation, pH, alkalinity and light during the competition process.

## Conclusions

Our experimental results call for a revision of the classic paradigm that cyanobacteria are superior competitors at low CO_2_ levels and high pH, whereas eukaryotic phytoplankton such as green algae are superior competitors at elevated CO_2_. Such simple dichotomies do not capture the diversity of CCMs that have been found among and within different phytoplankton taxa. First, our results demonstrate that green algae can also be very effective competitors at low CO_2_ levels. Second, our results show that some cyanobacterial strains are relatively poor competitors when CO_2_ is limiting but become stronger competitors at elevated CO_2_. We therefore urge the CCM scientific community to further elucidate and compare the performance of different CCMs, and their selective advantages and disadvantages across a wide range of different species at the molecular, physiological and ecological level. Such a comparative approach will be essential if we are to understand and predict how the species composition of natural phytoplankton communities will respond to the anticipated rise in CO_2_ levels.

## Supplementary data

Supplementary data are available at *JXB* online.

Model S1. Detailed description of the model.

Fig. S1. Dynamic changes in light, DIC and pH during the competition experiments.

Fig. S2. Dynamic changes in carbon speciation during the competition experiments.

Table S1. Steady-state characteristics in the monoculture experiments.

## Supplementary Material

Supplementary_Figures_S1_S2

Supplementary_Table_S1

Supplementary_Model

## Abbreviations:

CCM: CO_2_-concentrating mechanism
C_i_: inorganic carbon
CA: carbonic anhydrase
DIC: dissolved inorganic carbon
RuBisCO: ribulose-1,5-bisphosphate carboxylase/oxygenase
ppm: parts per million.
