# Supplementary material for: Competition between cyanobacteria and green algae at low versus elevated CO2: who will win, and why?
Source: J Exp Bot. 2017 Feb 16;68(14):3815–28. doi: 10.1093/jxb/erx027 (PMC5853874; doi:10.1093/jxb/erx027)
Supplement: Supplementary_Figures_S1_S2 [file erx027_suppl_supplementary_figures_s1_s2.pdf]

Supplementary Information

Competition between cyanobacteria and green algae at low versus elevated CO_2: who will win, and why?

Xing Ji, Jolanda M. H. Verspagen, Maayke Stomp and Jef Huisman

Legends of Figures S1 and S2

Fig. S1. Dynamic changes in light, DIC and pH during the competition experiments.

(A,B) Competition between *Monoraphidium* and *Scenedesmus*.

(C,D) Competition between *Monoraphidium* and *Chlorella*. (E,F) Competition between *Monoraphidium* and *Microcystis*. (G,H) Competition between *Microcystis* and *Scenedesmus*. (I,J) Competition between *Microcystis* and *Chlorella*. Left panels show experiments at low pCO_2 (100 ppm) and right panels at high pCO_2 (2,000 ppm). Symbols indicate experimental data, and lines indicate model predictions. Population dynamics of the competing species are in Fig. 4 of the main text. Parameter values of the model are provided in Tables 1 and 2.

Fig. S2. Dynamic changes in carbon speciation during the competition experiments.

(A,B) Competition between *Monoraphidium* and *Scenedesmus*. (C,D) Competition between *Monoraphidium* and *Chlorella*. (E,F) Competition between *Monoraphidium* and *Microcystis*. (G,H) Competition between *Microcystis* and *Scenedesmus*. (I,J) Competition between *Microcystis* and *Chlorella*. Left panels show experiments at low pCO_2 (100 ppm) and right panels at high pCO_2 (2,000 ppm). Symbols indicate experimental data, and lines indicate model predictions. Population dynamics of the competing species are in Fig. 4 of the main text. Parameter values of the model are provided in Tables 1 and 2.

Fig. S1

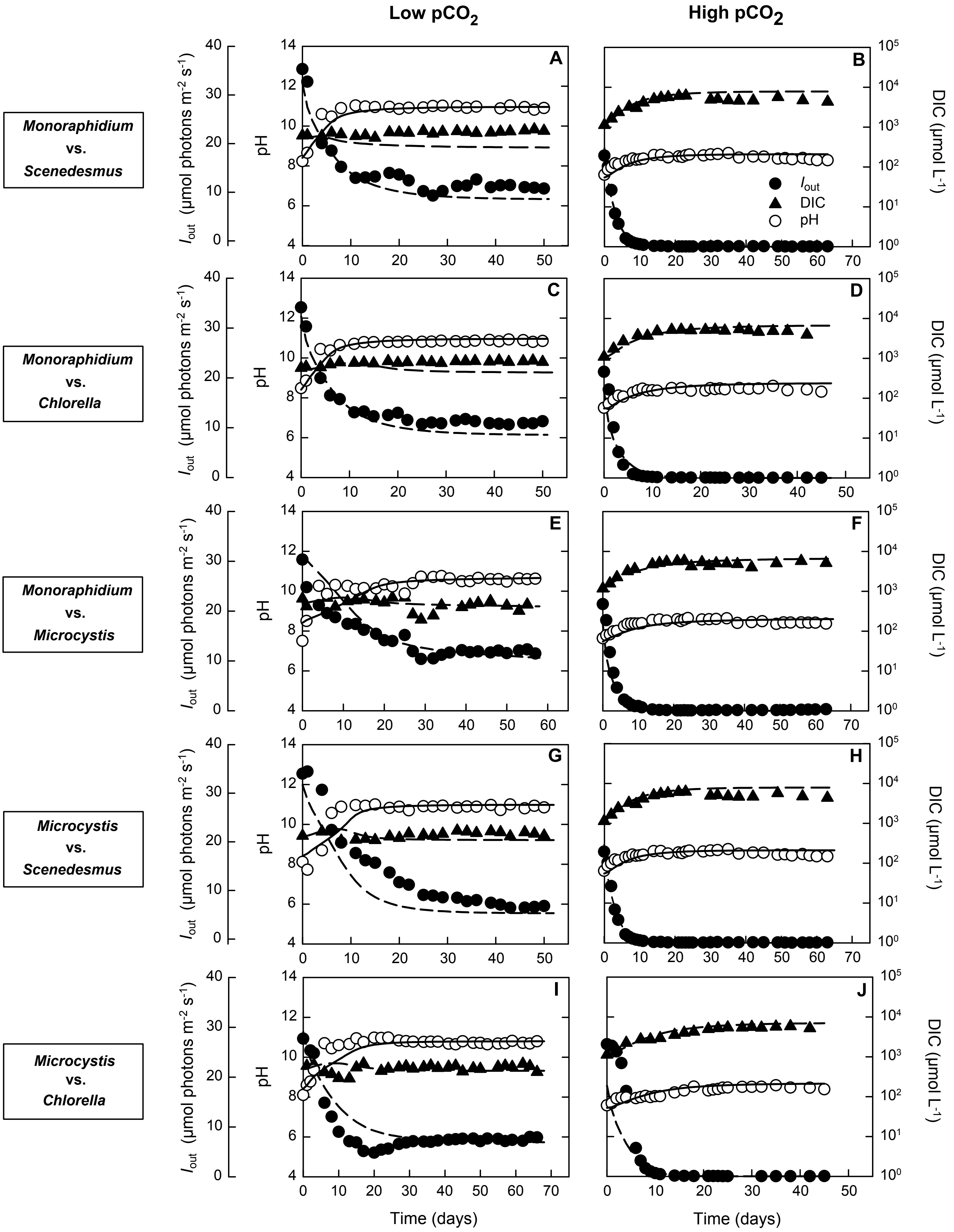

Low pCO2
High pCO2
Monoraphidium vs. Scenedesmus
Monoraphidium vs. Chlorella
Monoraphidium vs. Microcystis
Microcystis vs. Scenedesmus
Microcystis vs. Chlorella
A
B
C
D
E
F
G
H
I
J
Iout (µmol photons m-2 s-1)
pH
DIC (µmol L-1)
Iout
DIC
pH
Time (days)
Time (days)

Fig. S2

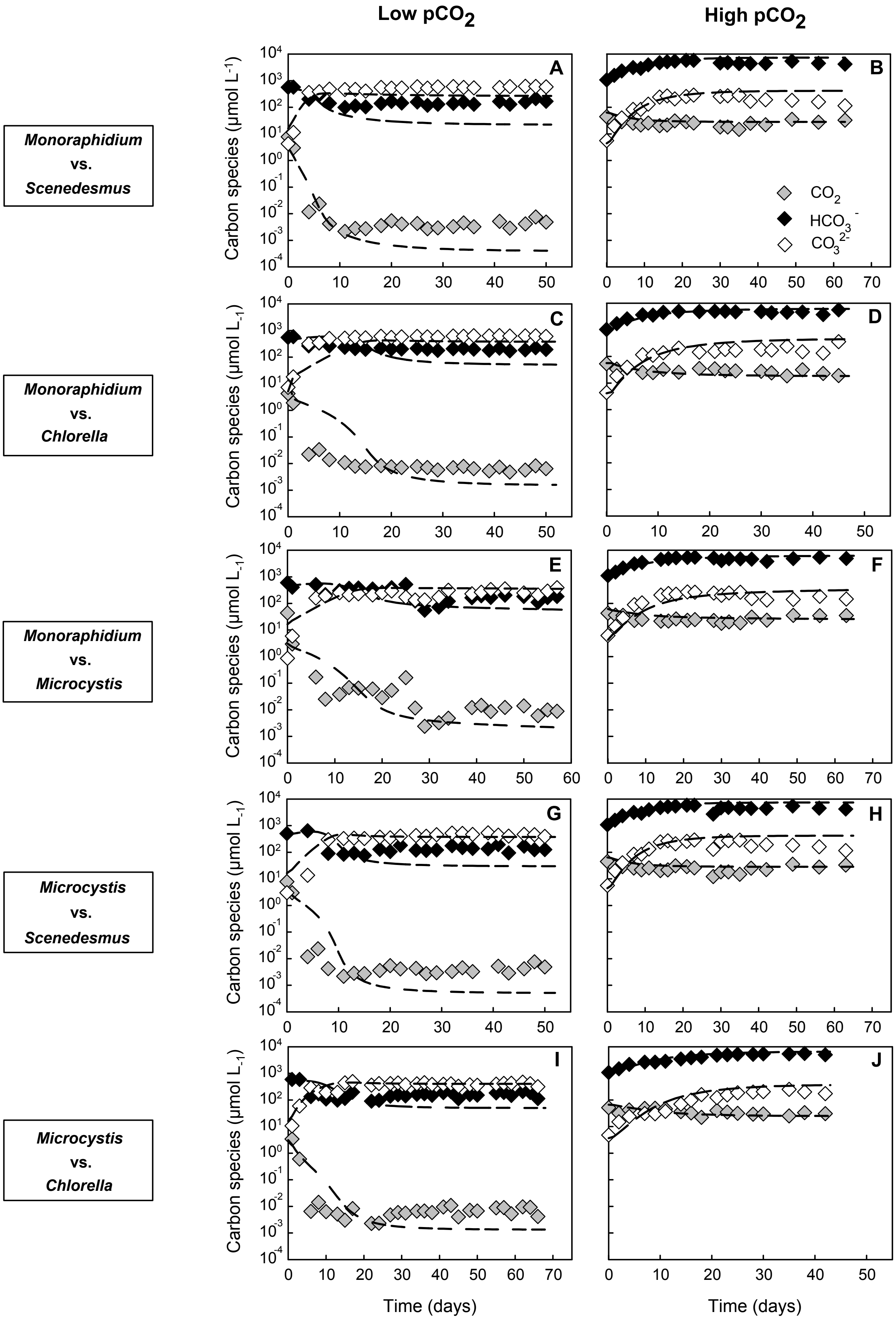

Low pCO2
High pCO2
Monoraphidium vs. Scenedesmus
Monoraphidium vs. Chlorella
Monoraphidium vs. Microcystis
Microcystis vs. Scenedesmus
Microcystis vs. Chlorella
A
B
C
D
E
F
G
H
I
J
CO2
HCO3 -
CO3 2-
Carbon species (µmol L-1)
Time (days)
