# Supplementary material for: Competition between cyanobacteria and green algae at low versus elevated CO2: who will win, and why?
Source: J Exp Bot. 2017 Feb 16;68(14):3815–28. doi: 10.1093/jxb/erx027 (PMC5853874; doi:10.1093/jxb/erx027)
Supplement: Supplementary_Table_S1 [file erx027_suppl_supplementary_table_s1.pdf]

## Supplementary Information

***Competition between cyanobacteria and green algae at low versus elevated $CO_2$: who will win, and why?***

*Xing Ji, Jolanda M. H. Verspagen, Maayke Stomp and Jef Huisman*

**Table S1.** Steady-state characteristics in the monoculture experiments.

| Parameter | *Monoraphidium* | *Scenedesmus* | *Microcystis* | *Chlorella* |
|---|---|---|---|---|
| Population density ($mm^3$ $L^{-1}$) | | | | |
| Low $pCO_2$ | 422 ± 21 | 722 ± 37 | 437 ± 12 | 370 ± 25 |
| High $pCO_2$ | 1943 ± 94 | 2556 ± 91 | 2103 ± 143 | 2353 ± 182 |
| Light penetration $I_{out}$ (μmol photons $m^{-2}$ $s^{-1}$) | | | | |
| Low $pCO_2$ | 7.89 ± 0.58 | 7.52 ± 0.38 | 6.24 ± 0.83 | 9.31 ± 0.29 |
| High $pCO_2$ | 0.15 ± 0.02 | 0.01 ± 0.00 | 0.01 ± 0.00 | 0.03 ± 0.01 |
| $CO_2$(aq) (μmol $L^{-1}$) | | | | |
| Low $pCO_2$ | 0.0048 ± 0.0013 | 0.0012 ± 0.0002 | 0.0041 ± 0.0017 | 0.0031 ± 0.0007 |
| High $pCO_2$ | 31.2 ± 2.5 | 30.4 ± 4.1 | 33.8 ± 2.9 | 29.9 ± 4.3 |
| $HCO_3^-$ concentration (μmol $L^{-1}$) | | | | |
| Low $pCO_2$ | 134 ± 16 | 49 ± 3 | 123 ± 25 | 84 ± 10 |
| High $pCO_2$ | 5714 ± 86 | 7391 ± 190 | 6008 ± 141 | 6492 ± 220 |
| pH | | | | |
| Low $pCO_2$ | 10.6 ± 0.2 | 10.8 ± 0.1 | 10.7 ± 0.1 | 10.7 ± 0.1 |
| High $pCO_2$ | 8.5 ± 0.0 | 8.6 ± 0.1 | 8.5 ± 0.0 | 8.6 ± 0.1 |
| Alkalinity (mEq $L^{-1}$) | | | | |
| Low $pCO_2$ | 2.01 ± 0.04 | 1.34 ± 0.10 | 1.97 ± 0.09 | 1.35 ± 0.18 |
| High $pCO_2$ | 6.24 ± 0.11 | 8.42 ± 0.18 | 6.51 ± 0.16 | 7.19 ± 0.22 |
| Cellular N:C ratio (molar ratio) | | | | |
| Low $pCO_2$ | 0.11 ± 0.01 | 0.14 ± 0.00 | 0.18 ± 0.01 | 0.13 ± 0.00 |
| High $pCO_2$ | 0.12 ± 0.00 | 0.14 ± 0.00 | 0.19 ± 0.01 | 0.14 ± 0.00 |

The steady-state values (± SD) are based on the last 5 time points measured in each of the monoculture experiments (i.e., *n*=5).
