# Supplementary material for: Competition between cyanobacteria and green algae at low versus elevated CO2: who will win, and why?
Source: J Exp Bot. 2017 Feb 16;68(14):3815–28. doi: 10.1093/jxb/erx027 (PMC5853874; doi:10.1093/jxb/erx027)
Supplement: Supplementary_Model [file erx027_suppl_supplementary_model.pdf]

Supplementary Information

Competition between cyanobacteria and green algae at low versus elevated CO_2: who will win, and why?

Xing Ji, Jolanda M. H. Verspagen, Maayke Stomp and Jef Huisman

Supplementary model. Detailed description of the model

Phytoplankton dynamics

Consider a number of i=1,…,n phytoplankton species. Let X_i denote the population density of phytoplankton species i, and let Q_i denote its cellular carbon content. The population dynamics of the competing phytoplankton species can then be written as a series of ordinary differential equations:

$$\frac{dX_i}{dt} = \mu_i(Q_i)X_i - m_iX_i \qquad i=1,\ldots,n \qquad \text{(A1)}$$

where $\mu_i(Q_i)$ is the specific growth rate of species i as an increasing function of its carbon content, and m_i is its loss rate.

Similar to previous models (Verspagen *et al.*, 2014a,b), we assume that carbon assimilated by phytoplankton is allocated to structural carbon biomass and a transient carbon pool. We define $Q_{min,i}$ as the minimum amount of cellular carbon that needs to be incorporated into the structural biomass of species i. Furthermore, we define $Q_{max,i}$ as the maximum amount of carbon that can be stored in its cell. The relative size of the transient carbon pool, T_i, is then given by:

$$T_i = \frac{Q_i - Q_{min,i}}{Q_{max,i} - Q_{min,i}} \qquad \text{(A2)}$$

Accordingly, the relative size of the transient carbon pool is constrained to $0 \leq T_i \leq 1$.

The transient carbon pool can be invested to make new structural biomass, which contributes to further phytoplankton growth. The specific growth rate of a species is therefore determined by the size of its transient carbon pool:

$$\mu_i(Q_i) = \mu_{max,i}\, T_i = \mu_{max,i}\left(\frac{Q_i - Q_{min,i}}{Q_{max,i} - Q_{min,i}}\right) \qquad \text{(A3)}$$

where $\mu_{max,i}$ is the maximum specific growth rate of species i.

This model formulation resembles Droop's (1973) classic growth model. However, Droop's growth equation considered only the minimum cellular quota $Q_{min,i}$. In our model, the cellular carbon quota are constrained between $Q_{min,i}$ and $Q_{max,i}$, as there are physical limits to the amount of carbon that can be stored inside a cell. The specific growth rate equals zero if the transient carbon pool is exhausted (i.e., $\mu_i = 0$ if $Q_i = Q_{min,i}$), and reaches its maximum if cells are satiated with carbon (i.e., $\mu_i = \mu_{max,i}$ if $Q_i = Q_{max,i}$).

Carbon uptake

The carbon contents of the phytoplankton species increase through uptake of carbon dioxide ($u_{CO2,i}$) and bicarbonate ($u_{HCO3,i}$), and decrease through respiration (r_i) and dilution of the cellular carbon content by growth:

$$\frac{dQ_i}{dt} = u_{CO2,i} + u_{HCO3,i} - r_i - \mu_i(Q_i)Q_i \qquad i=1,\ldots,n \qquad \text{(A4)}$$

Our model assumes that short-term uptake rates of carbon dioxide and bicarbonate are increasing functions of the ambient CO_2 and bicarbonate availability according to Michaelis-Menten kinetics. Since carbon uptake and assimilation require energy from the light reactions of photosynthesis, we further assume that the carbon uptake rates depend on photosynthetic activity. Finally, we assume that the carbon uptake rates decrease with an increasing size of the transient carbon pool (as in Verspagen *et al.*, 2014a,b). This provides a very simple negative feedback loop, such that the carbon uptake systems have the highest activity under carbon-limiting conditions and are down-regulated when cells are satiated with carbon. The uptake rates of CO_2 and bicarbonate by a phytoplankton species *i* can then be described by:

$$u_{CO2,i} = \left(\frac{u_{max,CO2,i}[\mathrm{CO_2}]}{H_{CO2,i}+[\mathrm{CO_2}]}\right)(1 - T_i)P_i \qquad \text{(A5)}$$

$$u_{HCO3,i} = \left(\frac{u_{max,HCO3,i}[\mathrm{HCO_3^-}]}{H_{HCO3,i}+[\mathrm{HCO_3^-}]}\right)(1 - T_i)P_i \qquad \text{(A6)}$$

where $u_{max,CO2,i}$ and $u_{max,HCO3,i}$ are the maximum uptake rates of CO_2 and bicarbonate, respectively, $H_{CO2,i}$ and $H_{HCO3,i}$ are the half-saturation constants, T_i is the relative size of the transient carbon pool (with $0 \le T_i \le 1$), and P_i is the relative photosynthetic activity of phytoplankton species i (with $0 \le P_i \le 1$).

Light availability determines the photosynthetic rate, and thereby the amount of energy available for carbon assimilation. We therefore calculate the relative photosynthetic activity of a phytoplankton species from its depth-averaged photosynthetic rate (Huisman and Weissing, 1994):

$$P_i = \frac{1}{z_{max}} \int_0^{z_{max}} p_i(\, I(z))dz \qquad \text{(A7)}$$

where p_i is the photosynthetic rate of species i, and z_{max} is the total depth of the water column. The notation $p_i(I(z))$ indicates that the photosynthetic rate is a function p_i of the local light intensity I, which in turn is a function of depth z.

The photosynthetic rate of a phytoplankton species is described by a simple Monod function of light intensity:

$$p_i(I) = \frac{p_{max,i} I}{H_{I,i} + I} \qquad \text{(A8)}$$

where $p_{max,i}$ is the maximum photosynthetic rate of species i, and $H_{I,i}$ is its half-saturation constant for light. The maximum carbon uptake rate is already accounted for in equations (A5) and (A6). Therefore, without loss of generality, we can set $p_{max,i} = 1$ (which constrains the depth integral in equation (A7) to $0 < P_i < 1$).

With the help of the Monod function (equation A8) and Lambert-Beer's law (equation 3 in the main text), the depth integral in equation (A7) can be solved analytically (Huisman and Weissing, 1994). This yields:

$$P_i = \left(\frac{1}{\ln(I_{in}/I_{out})}\right) \ln\left(\frac{H_{I,i} + I_{in}}{H_{I,i} + I_{out}}\right) \qquad \text{(A9)}$$

Carbon is lost by respiration. We assume that the respiration rate of species *i* is proportional to the size of its transient carbon pool:

$$r_i = r_{max,i} T_i \tag{A10}$$

where $r_{max,i}$ is the maximum respiration rate when its cells are fully satiated with carbon.

DIC, alkalinity and pH

Changes in the concentration of total dissolved inorganic carbon, [DIC], are described by (Verspagen *et al.*, 2014a):

$$\frac{d[\mathrm{DIC}]}{dt} = D([\mathrm{DIC}]_{in} - [\mathrm{DIC}]) + \frac{g_{CO2}}{z_{max}} - \sum_{i=1}^{n} \left(u_{CO2,i} + u_{HCO3,i}\right) X_i + \sum_{i=1}^{n} r_i X_i \tag{A11}$$

The first term on the right-hand side of this equation describes changes through the influx ($[\mathrm{DIC}]_{\mathrm{in}}$) and efflux of water containing DIC. The second term describes CO_2 exchange with the atmosphere, where g_{CO2} is the CO_2 flux across the air-water interface and division by z_{max} converts the flux per unit surface area into the corresponding change in DIC concentration. The third term describes uptake of dissolved CO_2 and bicarbonate by the photosynthetic activity of the phytoplankton community. Finally, the fourth term describes CO_2 release by respiration of the phytoplankton species.

The CO_2 flux across the air-water interface, g_{CO2}, depends on the difference in partial pressure. More specifically, g_{CO2} depends on the difference between the expected concentration of dissolved CO_2 in water if in equilibrium with the partial pressure in the atmosphere and the actual dissolved CO_2 concentration (Siegenthaler and Sarmiento, 1993; Cole *et al.*, 2010):

$$g_{CO2} = v(K_0 pCO_2 - [\mathrm{CO_2}]) \tag{A12}$$

where v is the gas transfer velocity (also known as piston velocity), K_0 is the solubility of CO_2 gas in water (also known as Henry's constant), pCO_2 is the partial pressure of CO_2

in the atmosphere, and $[CO_2]$ is the dissolved CO_2 concentration. In chemostats, gas transfer will depend on the gas flow rate (a). We therefore assume that $v = b\ a$, where b is a constant of proportionality.

Concentrations of dissolved CO_2, bicarbonate and carbonate can be calculated from [DIC] and pH (Stumm and Morgan, 1996). Changes in pH depend, in turn, on the alkalinity of water. In our application, alkalinity largely depends on the inorganic carbon and phosphate concentrations (Wolf-Gladrow *et al*., 2007):

$$ALK = [\mathrm{HCO_3^-}] + 2[\mathrm{CO_3^{2-}}] + [\mathrm{HPO_4^{2-}}] + 2[\mathrm{PO_4^{3-}}] + [\mathrm{OH^-}] - [\mathrm{H_3PO_4}] - [\mathrm{H^+}] \quad \text{(A13)}$$

Changes in dissolved CO_2 do not change alkalinity (see the above equation), and uptake of bicarbonate by phytoplankton requires the simultaneous uptake of a proton to maintain charge balance and hence does not change alkalinity either. However, assimilation of nutrients such as nitrate, phosphate and sulfate is accompanied by proton consumption to maintain charge balance and therefore increases alkalinity (Wolf-Gladrow *et al*., 2007). Our model therefore treats alkalinity as a dynamic variable:

$$\frac{dALK}{dt} = D(ALK_{in} - ALK) + \sum_{i=1}^{n}\left(u_{N,i} + u_{P,i} + 2u_{S,i}\right) X_i \quad \text{(A14)}$$

where ALK_{in} is the alkalinity of the influx, and $u_{N,i}$, $u_{P,i}$ and $u_{S,i}$ are the nitrate, phosphate and sulfate assimilation rates of phytoplankton species i. Nitrate and phosphate assimilation increase alkalinity by one mole equivalent, whereas sulfate assimilation increases alkalinity by 2 mole equivalents (Wolf-Gladrow *et al*., 2007). Accordingly, although nutrients do not limit phytoplankton growth in our model, the model does keep track of the nitrate, phosphate and sulfate concentration (see below) to calculate changes in alkalinity, and hence in pH and carbon speciation.

We used an iterative algorithm adapted from Portielje and Lijklema (1995) that, at each time step, calculates pH and the dissolved CO_2, bicarbonate and carbonate concentration from the [DIC] and alkalinity predicted by Eqs. (A11) and (A14). The algorithm is described in the Supporting Information of Verspagen *et al*. (2014a).

Nutrient assimilation

In our model application, nutrients do not limit phytoplankton growth. However, the model keeps track of nutrients such as phosphate, nitrate and sulfate, because the assimilation of these nutrients affects alkalinity and hence pH and carbon speciation (see above). For simplicity, we assume that the uptake rates of nitrate, phosphate and sulfate are proportional to the net uptake rate of carbon:

$$u_{j,i} = c_{j,i}\left(u_{CO2,i} + u_{HCO3,i} - r_i\right) \qquad \text{with } j = N,P,S \qquad \text{(A15)}$$

where $u_{N,i}$, $u_{P,i}$ and $u_{S,i}$ are the uptake rates of nitrate, phosphate and sulfate by species *i*, and $c_{N,i}$, $c_{P,i}$ and $c_{S,i}$ are the cellular N:C, P:C and S:C ratio of species *i*. Dynamic changes in the concentrations of dissolved inorganic nitrogen ([DIN]), phosphorus ([DIP]) and sulfur ([DIS]) can then be calculated from the uptake rates of the different species in the community:

$$\begin{aligned}
\frac{d[\mathrm{DIN}]}{dt} &= D\left([\mathrm{DIN}]_{\mathrm{IN}} - [\mathrm{DIN}]\right) - \sum_{i=1}^{n} u_{N,i} X_i \\
\frac{d[\mathrm{DIP}]}{dt} &= D\left([\mathrm{DIP}]_{\mathrm{IN}} - [\mathrm{DIP}]\right) - \sum_{i=1}^{n} u_{P,i} X_i \qquad \text{(A16)} \\
\frac{d[\mathrm{DIS}]}{dt} &= D\left([\mathrm{DIS}]_{\mathrm{IN}} - [\mathrm{DIS}]\right) - \sum_{i=1}^{n} u_{S,i} X_i
\end{aligned}$$

where $[\mathrm{DIN}]_{\mathrm{IN}}$, $[\mathrm{DIP}]_{\mathrm{IN}}$ and $[\mathrm{DIS}]_{\mathrm{IN}}$ are the concentrations of dissolved inorganic nitrogen, phosphorus and sulfur in the influx.

References

Cole JJ, Bade DL, Bastviken D, Pace ML, Van de Bogert M. 2010. Multiple approaches to estimating air-water gas exchange in small lakes. Limnology and Oceanography Methods **8,** 285-293.

Droop MR. 1973. Some thoughts on nutrient limitation in algae. Journal of Phycology **9,** 264-272.

Huisman J, Weissing FJ. 1994. Light-limited growth and competition for light in well-mixed aquatic environments: an elementary model. Ecology **75,** 507-520.

Portielje R, Lijklema L. 1995. Carbon dioxide fluxes across the air-water interface and its impact on carbon availability in aquatic systems. Limnology and Oceanography **40,** 690-699.

Siegenthaler U, Sarmiento JL. 1993. Atmospheric carbon dioxide and the ocean. Nature **365,** 119-125.

Stumm W, Morgan JJ. 1996. Aquatic chemistry: chemical equilibria and rates in natural waters. John Wiley & Son.

Verspagen JMH, Van de Waal DB, Finke JF, Visser PM, Van Donk E, Huisman J. 2014a. Rising CO_2 levels will intensify phytoplankton blooms in eutrophic and hypertrophic lakes. PloS One **9,** e104325.

Verspagen JMH, Van de Waal DB, Finke JF, Visser PM, Huisman J. 2014b.Contrasting effects of rising CO_2 on primary production and ecological stoichiometry at different nutrient levels. Ecology Letters **17,** 951-60.

Wolf-Gladrow DA, Zeebe RE, Klaas C, Körtzinger A, Dickson AG. 2007. Total alkalinity: the explicit conservative expression and its application to biogeochemical processes. Marine Chemistry **106,** 287-300.
